# Artificial Placental Respiration

**Published:** 1872-12-15

**Authors:** John Bartlett


### Original Communications.

### ARTIFICIAL PLACENTAL RESPIRATION.

At the meeting of the Chicago Society of Physicians and Surgeons, held Nov. 12th, a paper, avowedly a suggestion only, was read by Dr. John Bartlett, entitled, *Artificial Placental Respiration.*

Dr. Bartlett suggested, in certain cases in which the after-birth is extracted or extruded before the child, the following practice: *directly upon the detachment of the placenta, immerse it in the fresh, warm, defibrinated blood of some animal, as the sheep; change the blood as often as practicable, and aerate it by means of oxygen gas.*

Dr. Bartlett considered the statements going to show that the life of the fœtus was not momentarily dependent upon the purification of its blood through contact with the maternal circulation, too numerous to be disregarded. Three classes of cases in which the fœtus had for a considerable time survived a separation from the maternal circulation, were cited. In one class, the child maintained life in the dead body of the mother; in a second, the fœtus survived separated from the mother, its relations with the after-birth and membranes being retained; in the third, the child remained alive in utero, while the placenta was without the mother.

The circumstances and conditions under which the fœtus may, for a considerable time, continue its life after the disruption of its placental relations to the parent, seemed to be such as left the after-birth in situation, in some measure, to perform its branchial functions.

Dr. B. proceeded to point out by what simple processes of absorption and exhalation the embryo in the lower forms of vertebrata and in man in the earliest stages of embryonic existence, was nourished. He stated that the relation of the placenta of the chick to the albumen of the egg—its immersion in a fluid through which it absorbed oxygen and exhaled carbonic acid — was exactly such as he proposed to establish between the fœtus and the blood in which the placenta was immersed.

In regard to the probabilities of the simple immersion of the placenta in arterial blood proving adequate for the temporary respiration of the fœtus, the essayist called attention to the rapidity with which absorption took place through even the smallest abraded surfaces. The placenta presented fifty square inches of serous, and the like extent of fresh surface through which the necessary interchange of gases might take place. In referring to the anatomy of the placenta, the language of Owen was quoted: "The placental inter-communication is carried on by the contact of the fœtal capillaries with the *maternal extravasated blood.*" In this expression, Dr. Bartlett regarded his suggestion as foreshadowed.

Artificial placental respiration would prove available in those cases of placenta prævia in which the after-birth is detached, whether by the efforts of the uterus, or by the accoucheur. It might, also, offer an additional chance of resuscitation to the asphyxiated child, where the placenta is also delivered.

On account of the difficulty of obtaining blood of an inferior animal just when needed, Dr. Bartlett suggested that the addition of phosphate of soda and oxygen gas to water might render it a substitute. In some cases, the blood escaping from the mother might be made use of.

